# Patterns of engagement in care during clients’ first 12 months after HIV treatment initiation in South Africa: A retrospective cohort analysis using routinely collected data

**DOI:** 10.1371/journal.pgph.0002956

**Published:** 2024-02-28

**Authors:** Mhairi Maskew, Mariet Benade, Amy Huber, Sophie Pascoe, Linda Sande, Lufuno Malala, Musa Manganye, Sydney Rosen

**Affiliations:** 1 Health Economics and Epidemiology Research Office, Faculty of Health Sciences, University of the Witwatersrand, Johannesburg, South Africa; 2 Department of Global Health, Boston University School of Public Health, Boston, Massachusetts, United States of America; 3 Department of Medical Microbiology, Amsterdam University Medical Center, Amsterdam, The Netherlands; 4 HIV & AIDS Treatment, Care and Support Directorate, HIV & AIDS and STI Cluster, National Department of Health, Pretoria, South Africa; University of Washington, UNITED STATES

## Abstract

Retention on antiretroviral therapy (ART) during the early treatment period is one of the most serious challenges facing HIV programs, but the timing and patterns of early disengagement from care remain poorly understood. We describe patterns of engagement in HIV care during the first year after treatment initiation. We analysed retrospective datasets of routinely collected electronic medical register (EMR) data for ≥18-year-old clients who initiated ART at public sector clinics in South Africa after 01/01/2018 and had ≥14 months of potential follow-up. Using scheduled visit dates, we characterized engagement in care as continuous (no treatment interruption), cyclical (at least one visit >28 days late with a return visit observed) or disengaged (visit not attended and no evidence of return). We report 6- and 12-month patterns of retention in care and viral suppression. Among 35,830 participants (65% female, median age 33), in months 0–6, 59% were continuously in care, 14% had engaged cyclically, 11% had transferred to another facility, 1% had died, and 16% had disengaged from care at the initiating facility. Among disengagers in the first 6 months, 58% did not return after their initiation visit. By 12 months after initiation, the overall proportion disengaged was 23%, 45% were classified as continuously engaged in months 7–12, and only 38% of the cohort had maintained continuous engagement at both the 6- and 12-month endpoints. Participants who were cyclically engaged in months 0–6 were nearly twice as likely to disengage in months 7–12 as were continuous engagers in months 0–6 (relative risk 1.76, 95% CI:1.61–1.91) and were more likely to have an unsuppressed viral load by 12 months on ART (RR = 1.28; 95% CI1.13–1.44). The needs of continuous and cyclical engagers and those disengaging at different timepoints may vary and require different interventions or models of care.

## Introduction

As high HIV-prevalence countries in sub-Saharan Africa have neared global targets for HIV testing and for viral suppression among those remaining on treatment, the main challenge many countries face is retaining clients on antiretroviral therapy (ART) after treatment initiation. While South Africa continues to initiate large numbers of clients on ART—an average of some 366,000 per year between 2018 and 2022 [1]—engagement in ART care during a client’s first year after initiation, and in particular during the first six months, is especially problematic [2]. Two recent studies, one observational [3] and one a trial of a case management intervention [4], both estimated 6-month attrition from care at 26% in South Africa. Engagement during the early treatment period is essential both to achieving timely suppression of HIV viral load, which is required for both individual health and for reducing risk of ongoing transmission of HIV, and to establish the habit of medication adherence needed for lifelong treatment. In addition, the ART service delivery landscape in South Africa is changing, with new treatment guidelines introduced in 2023 recommending the first viral load test to be conducted after three one-month dispensing cycles (i.e. three months after treatment initiation). This effectively shifts eligibility for enrolment into differentiated care to as soon as the fourth month after ART initiation [5], rather than after 6 months as indicated by prior guidelines [6]. Good early engagement in care leading to timely viral suppression thus offers clients a chance to secure the benefits of less-intensive treatment delivery models [7] as early as possible after treatment initiation.

Although there is a large body of literature and multiple systematic reviews reporting rates of engagement and reasons for attrition from HIV treatment [8–13], the precise timing and patterns of client disengagement from care are poorly described. To address this problem, several previous studies have described standard care “trajectories” among ART clients [14–16]. These studies have largely reported retention and attrition in multi-year aggregate time periods, however, masking the details of client behavior during the two critical early periods, 0–6 and 7–12 months after initiation. In this study, we used retrospective medical record data from three provinces in South Africa to analyze patterns of care between ART initiation, 6 months after initiation, and 12 months after initiation. We used visit attendance to describe patterns of engagement in care and viral suppression during the first year on ART. This expands our outcomes beyond the typical retention variable, as our results show exactly when during these periods clients are disengaged from care and identify detailed patterns of behavior that can help target interventions to improve engagement in the first year of treatment.

## Methods

### Study populations and data

Sites for this study were public sector, primary healthcare clinics in Gauteng, Mpumalanga, and KwaZulu Natal (KZN) provinces in South Africa. Data were drawn from six facilities in West Rand District in Gauteng, six in Enhlanzeni District in Mpumalanga, and six in King Cetshwayo District in KZN. The six sites in each district, which comprise the sites currently participating in the SENTINEL study of differentiated service delivery [17], were selected because they were relatively large and captured diversity in facility setting (urban v rural) and in nongovernmental support partners. Access to data required both facility- and district-level Department of Health approvals, along with institutional ethics review and National Department of Health authorization.

For this study, we analyzed anonymized, retrospective cohort data from South Africa’s national electronic medical record system, Tier.net [18]. Individual, longitudinal HIV treatment records included site, date of ART initiation, observed dates of clinic visits and next scheduled clinic visit, sex, age at ART initiation, and observed dates of death or transfer to another facility. Scheduled visit dates reflected the healthcare provider’s determination of when a client should next interact with the healthcare system for a clinical consultation and/or medication refill, based on quantities of drugs dispensed at the facility. In this way, the next scheduled visit date should correspond with the end of the client’s medication supply, if the client has adhered to the expected daily dosing frequency and not obtained antiretroviral medications from any other source.

Inclusion criteria for the analytic sample were 1) observed ART initiation visit on or after 1 January 2018; and 2) ≥ 14 months of potential follow-up reflected in the dataset prior to the censor date for outcome variables. The 14-month endpoint provided a two-month window to observe visit attendance occurring after our 12-month observation period so that we could correctly classify visits scheduled at 12 months. Records from clients aged <18 or >85 years at date of ART initiation and records with a recorded ART initiation date but no observed visit dates in the datasets—including no visit on reported date of ART initiation—were excluded. This dataset allowed us to observe visit patterns for those who had previously initiated treatment, interrupted treatment, and then returned to the initiating facility after a period of disengagement. It did not, however, allow us to observe re-engagement in care at any facility other than the initiating one. Clients who disengaged from care at their initiating facility and then re-started treatment at a different facility—whether immediately or after any duration of interruption—appeared as permanent disengagers in our data set.

Dataset censoring dates were 13 July 2023 for Mpumalanga, 30 June 2023 for Kwa-Zulu Natal, and 6 July 2023 for Gauteng sites and represent the date of the latest visit observed in each of the datasets. Differences in censor dates reflect when the data were provided to the study team and which records and fields were made available to us in each district. All datasets capture visit history prior to, during and after the COVID-19 lockdown periods in South Africa. The data were accessed for research purposes on 10 August 2023 (Gauteng) and 17 August 2023 (Mpumalanga and Kwa-Zulu Natal).

Datasets were provided to the authors after all identifiers had been removed. The authors had no access to identifiable data for this analysis at any time.

### Variable definitions and outcomes

For this analysis, we use the term “engagement” to refer to a pattern of timely clinic visit attendance, in keeping with the usage in Ehrenkranz et al (2021) [19], which first proposed the notion of a “cyclical cascade.” For each individual participant in the dataset, we defined two sets of outcomes: 1) attendance at each visit after ART initiation; and 2) engagement patterns observed over 6-month intervals from 0–6 months and then 7–12 months after ART start. For the visit attendance outcomes, we used scheduled and observed visit dates to categorize attendance at every clinic visit after the ART initiation visit into one of three categories: 1) “attended as planned” where a visit was observed on or before the next scheduled date; 2) “attended late ≤28 days” for visits that were attended after but within 28 days of the scheduled date; and 3) “attended late >28 days” for visits that were attended more than 28 days after the scheduled date (Table 1). We used an interval of 28 days for consistency with U.S. President’s Emergency Program for AIDS Relief (PEPFAR) guidelines [20] and with South Africa’s 2023 Adherence Guidelines for HIV, TB and NCDs [21]. To account for visits that were scheduled but not attended at all, we also defined a “scheduled visit not attended” as the date at which disengagement was recognized for scheduled visits that were not ever attended during our observation period. The scheduled visit not attended date was defined for any particular client as 28 days after the last scheduled visit date.

**Table 1:** Visit types defined for study.

Visit category	Definition
Initiation visit	Observed date of ART initiation
Attended as planned	Visits attended on or before the next scheduled date
Attended late ≤ 28 days	Visits attended after but within 28 days of the scheduled date
Attended late ≥ 28 days	Visits attended more than 28 days after the scheduled date
Scheduled visit not attended	Date at which disengagement was recognized after a scheduled visit was missed. This date is 28 days after the last scheduled, unattended visit.

**Fig 1:**
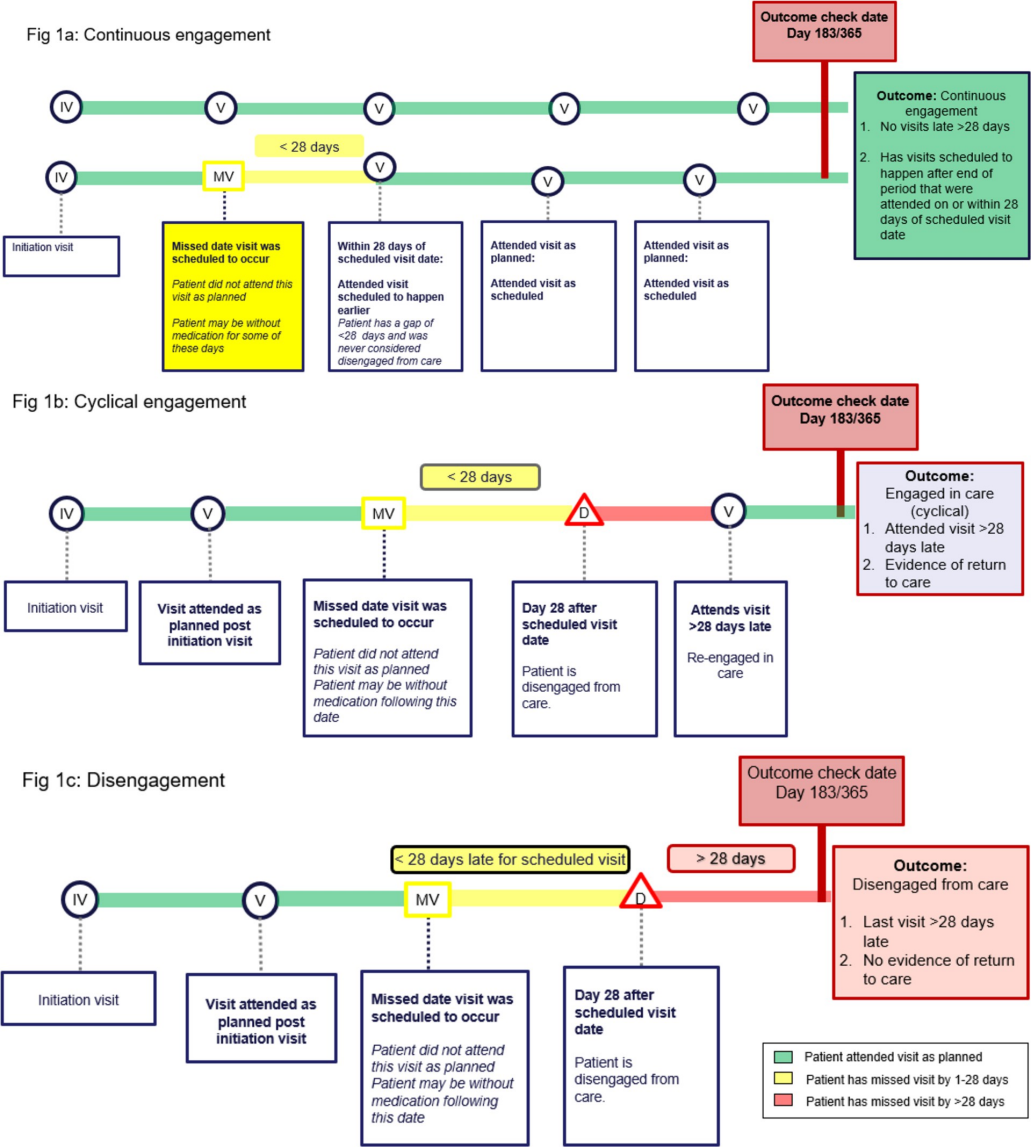
Engagement patterns and outcome definitions: 1) Continuous engagement; 2) Cyclical engagement; 3) Disengagement.

We then used these visit classifications to specify a set of engagement patterns for the periods from 0–6 months after initiation and from 7–12 months after initiation, as defined in Table 2 and summarized graphically in Fig 1. Client engagement was classified at these time points into one of three categories: continuously engaged in care (all visits attended and no visits were late >28 days), cyclically engaged in care (experienced treatment interruption but were in care at the end of the observation period), or disengaged from care. Disengagement was further categorized by the time at which disengagement was recognized, either immediately after initiation, within the first 3 months on ART, or between month 3 and 6 on ART. Participants who attended visits throughout the observation period and had a next scheduled visit date after 365 days were considered to be engaged in care at 12 months, even if no visit was observed in the data set after the 365-day mark.

**Table 2:** Patterns of engagement defined for study.

Label	Pattern of engagement observed during months 0–6 after ART initiation	Pattern of engagement observed during months 7–12 after ART initiation
Continuous	No visit > 28 days late in first 6 months and next visit scheduled >6 months after initiation (i.e. all visits as planned or late ≤28 days)	Continuous or cyclical at 6 months* AND no visit > 28 days late in months 7–12 and next visit scheduled >12 months after initiation (i.e., all visits attended as planned or late ≤28 days)
Cyclical	Attended at least 1 visit late by more than 28 days between initiation and month 6 but subsequently re-engaged in care by 6 months after initiation.	Continuous or cyclical at 6 months*; attended at least one visit late by more than 28 days between months 7 and 12 but subsequently re-engaged in care by 12 months after initiation.
Immediate disengagement	No visits after date of ART initiation; not observed during months 0–6 after ART initiation	Not reported for months 7–12; aggregated under “Disengaged months 0–6”
Early disengagement	≥1 visit after date of ART initiation but last visit ≤ 3 months after initiation	Not reported for months 7–12; aggregated under “Disengaged months 0–6”
Late disengagement	≥1 visit after date of ART initiation but last observed visit occurs 4–6 months after initiation (i.e. no further scheduled or observed visits in 7–12 month period)	Not reported for months 7–12; aggregated under “Disengaged months 0–6”
Disengaged months 0–6	Composite pattern including all immediate, early, or late disengagers in first 6 months; no visits observed after 6 months	Classified as immediate, early, or late disengagers in first 6 months AND no visits observed during months 7–12
Disengaged months 7–12	Not reported for months 0–6	Classified as continuous or cyclical at 6 months; at least 1 visit observed in months 7–12 but ≥1 scheduled visit late by >28 days with no evidence of return during months 7–14
Transferred	Documented transfer to another healthcare facility during month 0–6	Documented transfer to another healthcare facility at any time from 0–12 months
Died	Death recorded at any time from 0–6 months	Death recorded at any time from 0–12 months

*To be classified as either continuous or cyclical during months 7–12 after ART initiation, clients have been observed to be cyclical or continuous by month 6 after ART initiation. Those who disengaged from care during months 0–6 cannot be classified as continuous or cyclical thereafter, by definition, as their last observed visit occurred during the first 6 months on ART.

We then defined 12-month viral load (VL) outcomes using any VL results observed between 271 and 425 days (month 9-month 14) after ART initiation. For each observed test we classified the VL result as: 1) suppressed if VL result was <400 copies/mL; 2) viraemic episode if VL result was 400–1000 copies/mL; or 3) unsuppressed if VL result was >1000 copies/mL. We note that the current South African threshold for viral suppression is <50 copies/mL, but for this analysis we kept the previous threshold of <400 copies/mL which prevailed at the start of our observation period in 2018.

### Statistical analysis

We first reported cohort and participant characteristics at ART initiation using frequencies and simple proportions stratified by province. Next, we described the frequency and distribution of each defined visit type during the first 6 and 12 months after ART initiation as a proportion of the total monthly visits for that visit type, stratified by sex, age, district, year of ART initiation, CD4 at ART initiation, WHO classification at ART initiation, prior ART exposure, and TB status at initiation. We then reported the frequency and proportion of each of the engagement patterns defined at 6- and 12-months after ART initiation and characterize each pattern according to median number of visits and visit types during months 0–6 and 7–12 on ART. We illustrated these outcomes using an alluvial chart, which both depicts the first and second six-month periods after ART initiation and illustrates how participants appear to have shifted from one engagement pattern to another between the two time periods.

We investigated potential predictors of disengagement at both 6- and 12-months on ART using crude risk ratios and their corresponding 95% confidence intervals (CI). We also adjusted each estimate for other predictors using log-linear regression models to estimate adjusted risk ratios (aRR). We then estimated the effect of engagement pattern during the first 6 months on ART on the risk of disengagement during the months 7–12 on ART. As participants who had disengaged, transferred, or died during the first 6 months on ART could not be “newly” defined as disengaged during months 7–12, this analysis was restricted to the participants with engagement patterns characterized as continuous or cyclical at 6 months after starting ART. We conducted a crude analysis comparing the proportion of participants cyclically engaged at 6 months who disengaged from care by 12 months after ART initiation to the proportion of participants continuously engaged at 6 months achieving the same outcome by 12 months. We also conducted a crude analysis of 12-month VL results stratified by engagement pattern (continuous versus cyclical) at 6 months after ART initiation. For both of these outcomes, we estimated crude risk ratios and corresponding 95% confidence intervals and used log-binomial regression models to adjust estimates for potential confounding and present adjusted risk ratios and corresponding 95% CI.

### Outcome misclassification and sensitivity analysis

The data for this analysis are limited to within-facility observations; we could not track clients from one facility to another. It is thus likely that outcome misclassification bias may occur in our estimates due to “silent” transfers. In these instances, participants who did not attend a scheduled visit have not disengaged from care but are instead attending visits at another facility. Prior estimates indicate that outcome misclassification of this type may range from 8% [22] to 26% [23] within the context of ART treatment programs in the region. To quantify potential outcome misclassification in our estimates of the effect of 6-month engagement pattern (continuous or cyclical) on risk of disengagement by 12 months on treatment, we performed two sensitivity analyses, each at the extreme range of the published estimates of outcomes misclassification. For the first, we assumed equal rates of 12-month outcome misclassification for both the continuous and cyclically engaged participants at the lowest end of the range (8%). For the second, we assumed equal rates of 12-month outcome misclassification for both the continuous and cyclically engaged participants at the highest end of the range (26%). Crude estimates of the effect of engagement pattern at 6 months on risk of disengagement from care by 12 months on ART, accounting for potential outcome misclassification, are presented as relative risks with 95% confidence intervals for sensitivity analysis.

### Ethics statement

Analysis of the de-identified datasets was approved by the University of the Witwatersrand Human Research Ethics Committee (Medical) under protocol M1902105 and by the Boston University Medical Center IRB under protocol H-38815 for the use of data with a waiver of consent. In addition, permission to analyse these datasets were granted by the Mpumalanga Provincial Health Research and Ethics Committee (MP_202003_006), the KwaZulu-Natal Department of Health Research Committee (KZ_2020003_018), and the Ekurhuleni Health District Research Committee (GP_202003_034). All data were de-identified prior to sharing with the study teams and no direct participant contact occurred. Requirements for informed consent were waived by the ethics committees because data were de-identified.

## Results

### Description of study population

Our analytic sample included a total of 35,830 ART clients. Study site, demographic, and HIV treatment characteristics are shown in Table 3, by province and district. Cohorts were similar in terms of proportion female (between 64%-68%) and median age at ART initiation (between 31–33 years).

**Table 3:** Characteristics of study cohorts stratified by district (n = 35,830)*.

Characteristic	Mpumalanga cohort	KZN cohort	Gauteng cohort
District	Enhlanzeni	King Cetshwayo	West Rand
Total sample (n)	21,711	13,758	10,433
Dataset censoring date	13-Jul-23	30-Jun-23	6-Jul-23
Included in analytic sample (n, %)*	17,322 (77%)	10,753(78%)	7,755 (74%)
Number of study sites	6	6	6
Female (n, %)	11,021 (63.6%)	7,360 (68.4%)	5,226 (67.4%
Age at ART initiation			
Median (IQR)	32.0 [27.0, 39.0]	31.0 [25.0, 37.0]	34.0 [27.0, 41.0]
18–25	2,691 (25.0%)	1,382 (17.8%)	7,676 (21.4%)
26–49	12,319 (71.1%)	7,391 (68.7%)	5,668 (73.1%)
50+	1,400 (8.1%)	671 (6.2%)	705 (9.1%)
Year of ART initiation			
2018	3,099 (28.8%)	2,097 (27.0%)	8,834 (24.7%)
2019	5,225 (30.2%)	3,210 (29.9%)	1,906 (24.6%)
2020	4,275 (24.7%)	2,116 (19.7%)	1,615 (20.8%)
2021	3,123 (18.0%)	1,679 (15.6%)	1,628 (21.0%)
2022	1,061 (6.1%)	649 (6.0%)	509 (6.6%)
Setting			
Peri-Urban	5,544 (32.0%)	3,472 (32.3%)	0 (0%)
Rural	9,781 (56.5%)	3,513 (32.7%)	0 (0%)
Urban	1,997 (11.5%)	3,768 (35.0%)	7,755 (100%)
Facility Type			
Clinic	8,399 (48.5%)	10,753 (100%)	7,755 (100%)
Community Health Centre	8,923 (51.5%)	0 (0%)	0 (0%)
Baseline CD4**			
Median (IQR)	318 [174, 509]	362 [197, 552]	272 [134, 463]
CD4 <200 at baseline	3,497 (20.2%)	1,929 (17.9%)	1,649 (21.3%)
CD4 200+ at baseline	8,417 (48.6%)	5,686 (52.9%)	2,698 (34.8%)
No baseline CD4	5,408 (31.2%)	3,138 (29.2%)	3,408 (43.9%)
On TB treatment at ART initiation			
Yes	987 (5.7%)	721 (6.7%)	482 (6.2%)
No or no data	10325 (94.3%)	10,032 (93.2%)	7273 (93.7%)
HIV stage at initiation			
Stage 1	15,062 (87.0%)	8,435 (78.4%)	5,842 (75.3%)
Stage 2	1,192 (6.9%)	1,129 (10.5%)	1,002 (12.9%)
Stage 3	452 (2.6%)	482 (4.5%)	547 (7.1%)
Stage 4	81 (0.5%)	39 (0.4%)	109 (1.4%)
Unknown	535 (3.1%)	668 (6.2%)	255 (3.3%)
History of prior ART use			
None (naïve)	15,891 (91.7%)	10,060 (93.6%)	7,036 (90.7%)
Post exposure prophylaxis	5 (0.0%)	2 (0.0%)	0 (0%)
PMTCT	1,135 (6.6%)	620 (5.8%)	579 (7.5%)
PMTCT and Prior ART	57 (0.3%)	11 (0.1%)	5 (0.1%)
Prior ART >30 days	234 (1.4%)	60 (0.6%)	135 (1.7%)
ART regimen at initiation			
TDF/3TC/DTG	6,239 (36.0%)	3,232 (30.1%)	2,300 (29.7%)
TDF/FTC/EFV	10,817 (62.4%)	7,339 (68.3%)	5,374 (69.3%)
Other	266 (1.5%)	182 (1.7%)	81 (1.0%)
Number of clinic visits in first 12 months after initiation (Median [IQR])	7 [2, 9]	9 [5, 11]	7 [3, 9]
Time between initiation and last visit or disengagement (days) (Median [IQR])	375 [138, 408]	389 [242, 411]	382 [168, 411]

*Analytic sample was restricted to client records with ART initiation dates on or after 1 January 2018 and a minimum of 14 months’ potential observation time.

†Inclusive of the visit at which ART was initiated

**CD4 done 30 days prior, on, or within 30 days of day of ART initiation

PMTCT, prevention of mother-to-child transmission of HIV

The median number of visits observed during the first 12 months on ART, including the visit at which ART was initiated, was highest in the KZN sites (9 visits) and lowest in the Mpumalanga sites (7 visits); interquartile ranges were broad, from a low of just 2 visits to a high of 11.

### Distribution of visit types in first 6 and 12 months on ART

A total of 245,324 clinic visits were observed during the first 12 months on ART and classified into the five visit types shown in Table 1 (S1 Table). Most (62%) of the 122,382 visits that were attended after initiation in the first 6 months of care happened as planned, while more than a quarter were attended late but within 28 days. Only 4% of scheduled visits during this period were attended late by more than 28 days after the date scheduled, but 8% of scheduled visits were never attended at all. Similar results were observed when considering all visits during the first 12 months on ART.

As indicated in Table 4, the majority (60%) of 247,182 visits scheduled during the 12-month period after initiation were also attended as planned. A quarter (26.9%) of all first-year visits after initiation were late, but by less than 28 days. Only 5.3% were late by more than 28 days, while 8% were not attended at all. The proportion of scheduled visits not attended peaked sharply in month 2 after ART initiation at 19%, some three times higher than in any other month during the first year on ART (S1 Fig).

**Table 4:** Classification of visit types during the first six and twelve months after ART initiation, by engagement pattern.

**Visit**	**Engagement pattern months 0–6 after ART initiation**							
	*Continuous*	*Cyclical*	*Immediate disengagement*	*Early disengagement*	*Late disengagement*	*Transferred*	*Died*	*Total*
N (clients)	21,005 (58.6%)	4,967 (13.9%)	3,380 (9.4%)	539 (1.5%)	1,714 (4.8%)	3,813 (10.6%)	412 (1.1%)	35,830 (100%)
Number of visits per client in first 6 months after initiation (median, IQR)	5 [4, 6]	4 [2, 5]	1 [1, 1]	2 [2, 2]	3 [2, 4]	2 [1, 3]	2 [1, 3]	4 [2, 6]
Total visits in first six months after initiation*	91,065	12,936	3,371	1,103	5,097	7,971	839	122,382
Attended as planned	71.40%	43.30%	0%	34.70%	40.00%	36.10%	37.80%	62.30%
Attended late <28 days	28.60%	23.30%	0%	16.30%	21.40%	17.70%	16.60%	26.00%
Attended late >28 days	0%	33.40%	0%	0.10%	5.00%	3.60%	1.30%	4.00%
Scheduled visit in first 6 months not attended^#^	0%	0%	100%	48.90%	33.60%	42.50%	44.30%	7.70%
	**Engagement pattern months 7–12 after ART initiation**							
	*Continuous*	*Cyclical*	*Disengaged 0–6 months*	*Disengaged 7–12 months*	*n*.*a*.	*Transferred*	*Died*	*Total*
N (clients)	16,226 (45.3%)	5,741 (16.0%)	5,633 (15.7%)	2,334 (6.5%)		5,361 (15.0%)	535 (1.5%)	35,830 (100%)
Number of visits attended per client in first 12 months after initiation (median, IQR)	10 [8, 12]	8 [6, 10]	1 [1, 2]	5 [4, 6]		3 [1, 5]	2 [1, 5]	7 [3, 10]
Total visits in first 12 months after initiation*	160,106	46,056	10,292	13,409		15,925	1,398	247,182
Attended as planned	70.00%	51.80%	26.10%	45.70%		41.00%	44.10%	59.80%
Attended late <28 days	27.50%	29.90%	13.30%	26.80%		22.90%	19.70%	26.90%
Attended late >28 days	1.40%	18.60%	2.60%	8.40%		5.60%	4.40%	5.30%
Scheduled visit not attended^#^	0%	0%	58.00%	19.00%		30.60%	31.80%	8.00%

*See Table 1 for definition of visit types

^#^Not included for calculation of number of visits attended in row above

### Engagement patterns 6 and 12 months after ART initiation

**Fig 2:**
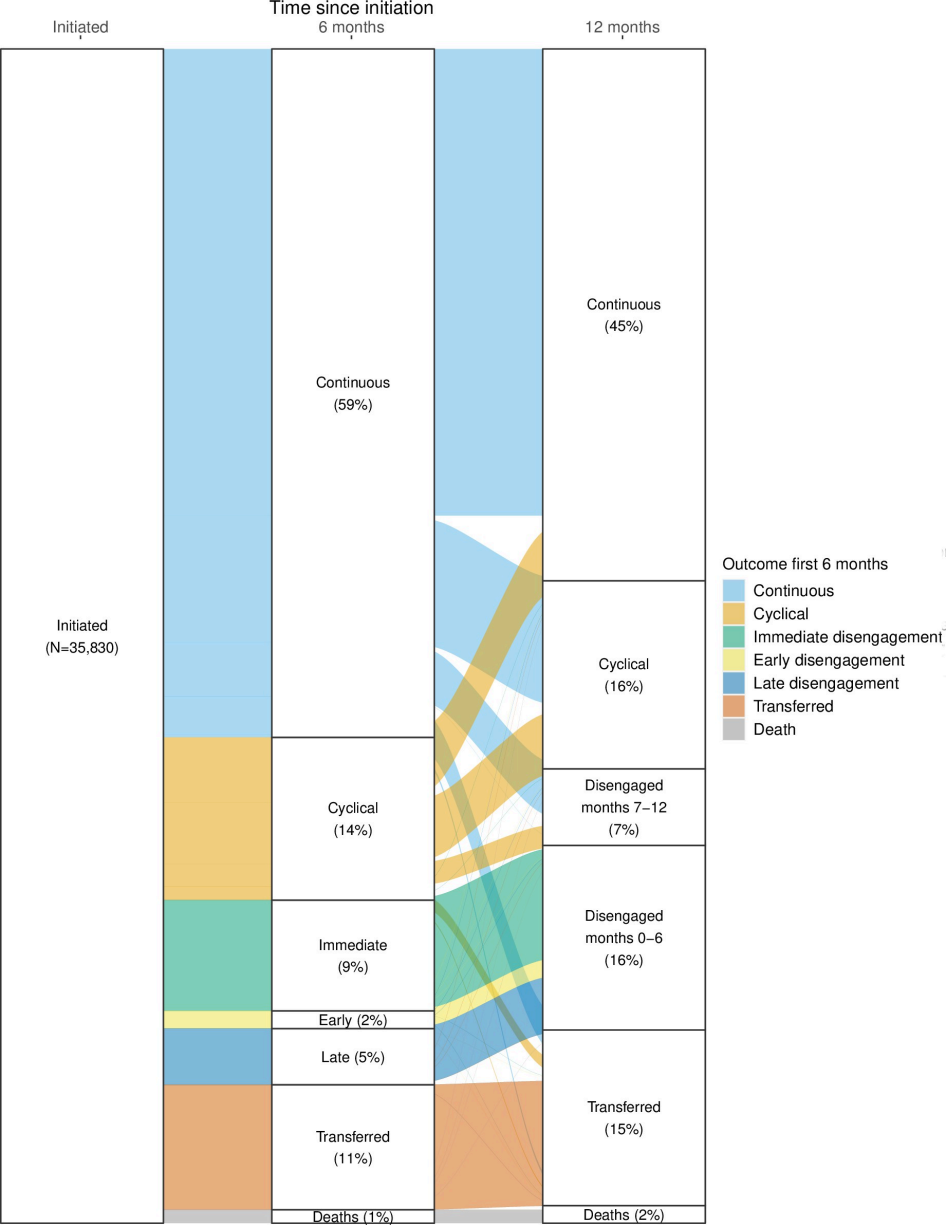
Engagement patterns at 6 and 12 months after ART initiation.

At the 6-month endpoint, 59% of the cohort remained continuously engaged, 14% showed a pattern of cyclical engagement, and 16% had disengaged from care (Fig 2 and Table 4). Among the 16% who disengaged from care, 59% disengaged immediately after ART initiation, 10% within the first 3 months on ART (early disengagers), and 31% between months 3 and 6 after initiation (late disengagers). Transfers occurred among 11% of the cohort, and the remaining 1% were reported to have died by 6 months after initiation. Participants who had continuously engaged in care at 6 months attended nearly three quarters (71%) of their visits as planned. Those with cyclical engagement attended fewer than half of their scheduled clinic visits as planned (43%), while those who disengaged from care during the first 6 months on ART attended only a third of visits as planned (34% among early disengagers and 40% among late disengagers).

Table 5 reports combined engagement patterns observed at 6- and 12-months after ART initiation; results are also illustrated in Fig 1. At 12 months after initiation, most of those who were continuously engaged at 6 months (67.8%), stayed in that category, but a substantial minority (18.4%) shifted to a cyclical engagement pattern, while others (7.8%) disengaged from care during this period. Most who were cyclically engaged during the first six months shifted to continuous care (40%), with some remaining cyclical in their engagement (37.7%) and some becoming disengaged (13.8%). At the 12-month endpoint, a total of 45.3% had been continuously engaged throughout the period from months 7 to 12, 16% ended as cyclical engagers, 15% had transferred to another facility, 1.5% had died, and 6.5% fell into the 7-12-month disengagement category.

**Table 5:** Engagement patterns 6 and 12 months after ART initiation.

**12 month pattern**	**Engagement pattern months 0–6 after ART initiation**							
	*Continuous*	*Cyclical*	*Immediate disengage-ment*	*Early disengage-ment*	*Late disengage-ment*	*Transferred*	*Died*	*Total*
	21,005 (58.6%)	4,967 (13.9%)	3,380 (9.4%)	539 (1.5%)	1,714 (4.8%)	3,813 (10.6%)	412 (1.1%)	35,830 (100%)
	↓	↓	↓	↓	↓	↓	↓	↓
**Engagement pattern months 7–12 after ART initiation, by 6-month pattern**								
*Continuous*	14,238 (67.8%)	1,988 (40.0%)	0 (0%)	0 (0%)	0 (0%)	0 (0%)	0 (0%)	16,226 (45.3%)
*Cyclical*	3,866 (18.4%)	18,75 (37.7%)	0 (0%)	0 (0%)	0 (0%)	0 (0%)	0 (0%)	5,741 (16.0%)
*Disengaged* *months 0–6*	0 (0%)	0 (0%)	3,380 (100%)	539 (100%)	1,714 (100%)	0 (0%)	0 (0%)	5,633 (15.7%)
*Disengaged* *months 7–12*	1,648 (7.8%)	686 (13.8%)	0 (0%)	0 (0%)	0 (0%)	0 (0%)	0 (0%)	2,334 (6.5%)
*Transferred*	1,162 (5.5%)	386 (7.8%)	0 (0%)	0 (0%)	0 (0%)	3,813 (100%)	0 (0%)	5,361 (15.0%)
*Died*	91 (0.4%)	32 (0.6%)	0 (0%)	0 (0%)	0 (0%)	0 (0%)	412 (100%)	535 (1.5%)

By one year after initiation, only 39.7% of the cohort had maintained continuous engagement for the full 12-month period. An additional 5.2% had engaged cyclically for the entire year. 16.3% shifted between continuous and cyclical engagement over the course of the year. Overall disengagement during the first year totaled 22.2%.

### Patterns of engagement by province, year of initiation, age, and gender

Patterns of engagement in the first 6 months of care differed slightly between provinces. KwaZulu-Natal had the lowest proportion of client disengagement at 10%, compared to 16% in Mpumalanga and 22% in the Gauteng district (2). Differences by province followed similar trends for months 7–12 on ART as in the first 6 months. Disengagement between months 7 to 12 was highest in Gauteng (9%), followed by Mpumalanga (7%) and KwaZulu-Natal (5%). Cyclical engagement was higher in Gauteng (16%) and KwaZulu-Natal (20%) than in Mpumalanga (14%). The number of visits per participant was highest in KwaZulu-Natal (9, IQR 5–11), followed by Gauteng (7, ICR 3–9) and Mpumalanga (7, IQR 2–9), respectively.

Annual trends in the patterns of engagement also differed between provinces. Proportions defined as disengaging from care at 6 months increased in 2020 and 2021 during the COVID-19 pandemic (S2 Table). In Mpumalanga, disengagement at 6 months increased from 12% among participants initiated in 2019 to 18% in 2020 and 2021 and 21% in 2022. Rates of cyclical engagement in Mpumalanga increased by 50% between 2019 (10%) and 2022 (15%). In KwaZulu Natal, however, continuous engagement in care increased from 51% in 2018 to 64% in 2019 and 69% in 2020, after which it decreased to 65% and 63% in 2021 and 2022, respectively. In Gauteng, continuous engagement hovered around 60% over the study period (2018–2022).

No meaningful differences in engagement pattern were observed between females and males during either the first or second 6-month periods on ART (S3 Table). While the number of participants who died was low (n = 535), we found that those who died were older, more likely to be men, have a CD4 count <200 at initiation, and be on TB treatment compared to other clients. Year of ART initiation had no discernible impact on risk of death (S4 Table). Deaths remained stable or were lower at both end points in 2020 compared to earlier and later years, despite the COVID-19 pandemic (S2 Table).

Age was strongly associated with differences in engagement profile (Table 6). The proportion of participants engaged in a continuous pattern of care during the first 6 months rose from 50% among participants aged 18–25 to 60% among those aged 25–49 years to 66% for those aged ≥50 years. Participants aged 18–25 years were most likely to be engaged in a cyclical pattern (17% compared to 13% (26–49) and 11% (≥50 years)) or have transferred to a different facility (14% compared to 10% and 9%, respectively). Increased age was associated with a higher proportion of participants dying in the first year after ART initiation (0.4% among 18–25 vs 3.1% for those aged ≥50 years). Patterns of engagement in months 7–12 also indicated differences by age group. Younger participants (aged 18–25 years) were less likely to be engaged in continuous care than those aged 25–49 years and those ≥50 years (36% vs 47% and 57% respectively). Those aged 18–25 years were also more likely to be engaged in cyclical care (17% vs 16% and 13%), to have disengaged from care (27% vs 22% and 15%), or to have transferred (20% vs 14% and 12%) than those age 25–49 years and those ≥50 years, respectively. Participants aged 18–25 also attended fewer visits (6; IQR 2–9) than did their older counterparts (26–49 years: 7 visits (IQR 3–10); ≥50 years: 8 visits (IQR 4–10)).

**Table 6:** Classification of visit types and engagement patterns during the first and second six months after ART initiation stratified by age group.

Outcome	18–25 years	25–49 year	50+ years	Overall
N	7,676	25,378	2,776	35,830
**Outcome in months 0–6**	** **	** **	** **	** **
Continuous	3,828 (49.9%)	15,340 (60.4%)	1,837 (66.2%)	21,005 (58.6%)
Cyclical	1,264 (16.5%)	3,392 (13.4%)	311 (11.2%)	4,967 (13.9%)
Immediate disengagement	839 (10.9%)	2,350 (9.3%)	191 (6.9%)	3,380 (9.4%)
Early disengagement	146 (1.9%)	362 (1.4%)	31 (1.1%)	539 (1.5%)
Late disengagement	487 (6.3%)	1,142 (4.5%)	85 (3.1%)	1,714 (4.8%)
Transferred	1,082 (14.1%)	2,495 (9.8%)	236 (8.5%)	3,813 (10.6%)
Died	30 (0.4%)	297 (1.2%)	85 (3.1%)	412 (1.1%)
**Outcome in months 7–12**	** **	** **	** **	** **
Continuous	2,736 (35.6%)	11,918 (47.0%)	1,572 (56.6%)	16,226 (45.3%)
Cyclical	1,337 (17.4%)	4,055 (16.0%)	349 (12.6%)	5,741 (16.0%)
Disengaged 7–12	1,472 (19.2%)	3,854 (15.2%)	307 (11.1%)	5,633 (15.7%)
Disengaged 0–6	568 (7.4%)	1,650 (6.5%)	116 (4.2%)	2,334 (6.5%)
Transferred	1,518 (19.8%)	3,525 (13.9%)	318 (11.5%)	5,361 (15.0%)
Death	45 (0.6%)	376 (1.5%)	114 (4.1%)	535 (1.5%)
Number of visits (median, IQR)	6 [2, 9]	7 [3, 10]	8 [4, 10]	7[3, 10]
Time between initiation and last visit or disengagement (median, IQR)	371 [123, 405]	384 [184, 411]	390 [251, 414]	381 [169, 410]

### Predictors of outcomes

As noted above, age was strongly associated with risk of disengagement from care in the first and second 6-month periods on ART (S5 Table). Participants aged 18–25 years were 1.73 (95% CI: 1.54–1.94) times more likely to disengage than those older than 50 years, while those aged 26–49 were 1.37 (1.23–1.53) times more likely to be disengaged at month 6. Differences in risk of disengagement by age remained after adjusting for gender, year of ART initiation, and district, with those aged 18–25 being nearly twice (RR 1.85, 95% CI: 1.65–2.08) as likely to be disengaged and those aged 26–40 nearly at 50% (RR 1.43, 95% CI: 1.28–1.59) higher risk of being disengaged at 6 months when compared to those older than 50 years. Participants who initiated in 2019 had lower risk of disengagement in the first 6 months (crude RR: 0.66) compared to 2018. This finding persisted when adjusting for age, district, and gender (2019 adjusted RR: 0.74, 95% CI: 0.69–0.79).

When considering disengagement between months 7–12, those who were engaged cyclically in months 0–6 had 1.76 (95% CI: 1.62–1.91) times the risk of becoming disengaged in months 7–12 compared to those who were continuously engaged in months 0–6. This pattern persisted when adjusting for age, gender, year of initiation, and district (adjusted RR 1.75; 95% CI: 1.61–1.91). Age was also associated with higher risk of disengagement: those aged 18–25 years were about twice (crude RR: 2.06 (95% CI: 1.70–2.51) adjusted RR: 2.09 (95% CI: 1.75–2.59) as likely as those older than 50 years to become disengaged.

### Impact of 6-month engagement pattern on 12-month viral suppression

We then restricted analytics to those with a continuous (n = 21,005) or cyclical (n = 4,967) engagement pattern at 6 months and stratified viral load results observed between 9 and 14 months on ART by 6-month engagement pattern (Table 7). We noted that, overall, cyclical engagers were less likely to have VL results observed and also less likely to have a suppressed VL at 12 months compared to those continuously engaged in care during the first 6 months of treatment. When we further restricted the analysis to those with observed VL results, rates of unsuppressed VL results were somewhat higher among cyclical engagers compared to those continuously engaged in care at 6 months on ART.

**Table 7:** Viral load suppression at 12 months on ART stratified by 6-month engagement pattern (n = 25,972).

Viral load result in month 9-month 14*	Continuous engagement 0–6 months (N = 21,005)	Cyclical engagement 0–6 months (N = 4,967)	Crude relative risk (95% CI)	Adjusted relative risk (95% CI)
VL suppressed (<400 copies/mL)	12,425 (60%)	2,243 (45%)	0.76 (0.74–0.79)	0.77 (0.75–0.80)
VL unsuppressed (>1000 copies/mL)	1,056 (5%)	325 (7%)	1.30 (1.15–1.47)	1.28 (1.13–1.44)
Viremic episode (400–1000 copies/mL)	296 (2%)	55 (1%)	0.79 (0.59–1.05)	0.81 (0.61–1.09)
Missing (VL not done)	7,228 (34%)	2,344 (47%)	1.37 (1.32–1.42)	1.35 (1.30–1.39)

*Note: As explained above, the current threshold for viral suppression in South African guidelines is <50 copies/ml, rather than the previous threshold of <400 copies used here [21].

### Sensitivity analysis

In sensitivity analysis, we observed the impact of potential outcome misclassification on the estimates of risk of disengagement due to silent transfer (S6 Table). The sensitivity analysis showed that under the assumption of non-differential misclassification (misclassification of outcomes happened in equal proportions to both the continuous engagers and cyclical engagers), our original analysis would underestimate the risk of disengagement by less than 20 percentage points regardless of the extent of misclassification.

## Discussion

In this study of more than 35,000 clients initiating HIV treatment in four districts in South Africa, fewer than 60% and 45% of clients were continuously engaged in care (no interruptions >28 days) at 6 and 12 months after treatment initiation, respectively, at their initiating facilities. Cyclical engagement, a pattern of remaining in care but being late for visits, suggesting interruptions in medication possession, was common, experienced by some 14% of clients even in their first six months. Nearly 90% of participants who disengaged from care during the first 6 months on ART did so either immediately after the initiation visit or within the first three months on ART. Almost a quarter of clients in the cohort (23%) had disengaged from ART by the end of the first year.

Continuity of treatment remains a critical objective in ending the HIV epidemic. Despite huge advances in access to ART in the era of UTT, current models of HIV service delivery still struggle to achieve high levels of sustained engagement in care after treatment initiation, particularly in the early treatment period. Our results suggest that not only is the first 6 months on ART a high-risk period for disengagement from care; it also appears to be critical for establishing future patterns of engagement with HIV care programs. For the most part, patterns of engagement that were established during the first 6 months on ART demonstrated little change in months 7–12. Cyclical and disengagement outcomes by 6 months on ART, moreover, were frequently preceded by multiple occurrences of visits attended late, suggesting opportunities to intervene with clients at risk of disengagement may be missed in the early ART period if visit patterns are not recognized. Given how many of the clients (58%) who disengage from care in the first six months do so immediately after ART initiation, waiting for a second visit to intervene is too late.

The six-month outcomes we observed are consistent with those reported by others in South Africa and in the region more broadly. A systematic review of engagement on HIV treatment estimated an average 6-month engagement rate of 85% for studies from in South Africa [24], while a more recent analysis of routinely collected data reported that 74% of participants in South Africa were still engaged at 6 months after initiation [3]. Our study adds to the existing literature by examining the early treatment period in a level of detail not previously reported and relating patterns of engagement to visit behavior. One exception to the consistency of engagement patterns over the first year on ART was observed among those with a pattern of cyclical engagement in care. Despite not fully disengaging from care, participants with cyclical patterns of care were less likely to attend scheduled visits as planned compared not only to those engaging in care continuously but to those who fully disengaged from care within the first 6 months on treatment. In addition, clients not in care are less likely to receive services required for disease management such as viral load testing. Indeed, cyclical engagers were less likely to have a viral load done during the first year on ART compared to their counterparts who were continuously engaged in care and therefore had lower documented rates of viral suppression as a group. As mentioned above, irregular visit attendance likely means at least some periods of treatment interruption during this critical period on ART, potentially compromising the client’s own health [25] and creating opportunities for ongoing transmission of HIV that can be prevented with sustained ART [26]. While interruptions are increasingly being seen as “normal” for lifelong chronic disease treatment [19], they are still not desirable, particularly before an ART client has achieved sustained viral suppression [25].

Cyclical engagement as we observe it in these datasets may reflect new initiators’ need for greater flexibility in access to care, as has been demonstrated by differentiated service delivery (DSD) models throughout the region [27–29]. In South Africa, as in many other countries, however, eligibility for access to differentiated models of care has historically been limited those already on ART for at least 6 months [2]. Those in the first 6 months continue to be required to make multiple clinic visits and given just 1 or 2 months’ supplies of medications at a time. Numbers of visits required in national guidelines across the region vary [30], but individual facilities may still ask for multiple visits during the early treatment period. Although South Africa currently requires only four visits during the first six months, the sites in this study informed study staff that during the study period they asked clients to come to the clinic an average of 7 or 8 times during this period, including the visit at which treatment is initiated. We have found similar practices in Zambia, another high HIV-prevalence southern African country facing challenges with engagement in care [31].

In our study, nearly 30% of individuals who demonstrated cyclical patterns of engagement during the first 6 months on ART were observed to engage continuously during months 7–12, a period when they were eligible for less burdensome DSD models such as community-based medication pickups. In recognition of the potential value of earlier eligibility for DSD models, in April 2023 the South African National Department of Health announced that going forward, viral load tests will be performed after 3 months on ART rather than 6, and clients with viral suppression will be eligible for enrollment in DSD models at the 4-month point [5]. While this will not immediately affect the very early disengagers seen in our study, it may encourage some to persist in care for the short period until they are eligible. The April 2023 guidelines also reduced the number of clinic visits required as part of the treatment initiation process and introduced procedures for clients re-engaging in care, changes that may also help improve overall retention in care.

We observed the lowest rates of continuous engagement in care among youth aged 18–25 years, with less than half continuously engaged in care at 6 months and only a third continuously engaged by 12 months. Concerning rates of disengagement form care on ART among adolescents and youth have been described previously [32–36]. Despite implementation of models of care designed to address engagement challenges in this group, such as school and youth adherence clubs, youth engagement remains sub-optimal. Understanding reasons for disengagement among young adults will be critical if we are to design effective interventions that can affect patterns of engagement from the start of HIV treatment [37].

Our results should be considered within the context of the limitations relevant to observational cohorts comprised from routinely collected EMR sources. First, the datasets analyzed here do not observe clinic attendance outside of the facilities at which participants initiated ART and are thus not robust to silent transfers. While tracing clients disengaged from care is likely the most accurate method to ascertain vital and care status [38, 39], it is not possible to do this with the EMR data used in this study. Rather than speculate on the possible impact of this type of outcome misclassification, we performed sensitivity analyses considering scenarios of both differential and non-differential misclassification of disengagement from care. The results suggest that non-differential misclassification (more likely in this context as both exposure and outcome variables were assessed independently of each other) would result in underestimation of the risk of disengagement from care: even in the presence of poor sensitivity of outcome misclassification, the estimate of the effect of pattern of engagement at 6 months on ART on subsequent disengagement in care was likely biased only minimally towards the null.

Second, as we noted previously, visit attendance is used here as a proxy for medication possession. Medication possession may not directly correlate to medication adherence, however. To the extent to which visit attendance was more consistent than medication adherence, our estimates would over-estimate engagement in care. On the other hand, clients may still have medications in hand for some period of time after missing a clinic visit, in which case our estimates would be an underestimate of continuous engagement. In addition, the EMR datasets we used may not observe medication pickups at external pickup points, which may have been utilized by some participants during the second 6 months on ART and may also have occurred without documentation during the first 6 months on ART during the COVID-19 pandemic, leading to an overestimate of 6-month disengagement in our results during that period (2020–2022). Restrictions on transport, clinic closures, and other COVID-19 pandemic measures likely did deter clinic visits in the public sector starting in March 2020, however, such that observed engagement patterns may have been accurate even if they do not reflect non-pandemic trends. While using scheduled visit dates in relation to observed visits should, at least in part, mitigate this limitation, we cannot be sure that this occurred consistently and universally across all sites and datasets. Misclassification like this could result in overestimation of disengagement during months 7–12.

Despite these limitations, our results highlight important considerations for models of HIV service delivery during the early treatment period. We demonstrate clearly distinct patterns of engagement in HIV care during the early treatment period and their associated risk of subsequent treatment interruption. It is likely that the needs of continuous and cyclical engagers differ from those who disengage from care during the first 6 months after initiation and require different interventions or models of care. Qualitative research with persons with both continuous and cyclical patterns of engagement may reveal potentially modifiable barriers to improving outcomes in the early treatment period and beyond. Strengthening facility-level interventions such as the counselling offered at treatment initiation and the implementation of the “adherence plans” required by South Africa’s national guidelines may also help improve outcomes [21].

## Supporting information

S1 Table Distribution of visit types during the first 12 months on ART. (DOCX)

S2 Table Classification of engagement profile by district and year of initiation. (DOCX)

S3 Table Classification of visit types and engagement patterns during the first and second six months after ART initiation, stratified by gender. (DOCX)

S4 Table Characteristics of participants by engagement pattern months 7–12. (DOCX)

S5 Table Crude and adjusted predictors of becoming disengaged from care. (DOCX)

S6 Table Sensitivity analysis adjusting for potential outcome misclassification. (DOCX)

S7 Table ART regimen by year of initiation. (DOCX)

S1 Fig Distribution of last treatment visits by month after initiation. (DOCX)

S1 File Inclusivity in global research questionnaire. (DOCX)
