# Supplementary material for: Patterns of engagement in care during clients’ first 12 months after HIV treatment initiation in South Africa: A retrospective cohort analysis using routinely collected data
Source: PLOS Glob Public Health. 2024 Feb 28;4(2):e0002956. doi: 10.1371/journal.pgph.0002956 (PMC10901315; doi:10.1371/journal.pgph.0002956)
Supplement: S1 Table — (DOCX) [file pgph.0002956.s001.docx]

**S1 Table: Distribution of visit types during the first 12 months on ART**

| Month | As planned |  | Late <28 days | | Late >28 days | | Scheduled visits not attended | | Total Visits |
| --- | --- | --- | --- | --- | --- | --- | --- | --- | --- |
| Initiation |  |  |  |  |  |  |  |  | 35830 |
| 1 | 20058 | 70% | 7697 | 27% | 121 | 0% | 604 | 2% | 28480 |
| 2 | 13063 | 54% | 5442 | 23% | 944 | 4% | 4617 | 19% | 24066 |
| 3 | 14030 | 63% | 5878 | 26% | 1075 | 5% | 1279 | 6% | 22262 |
| 4 | 11425 | 61% | 4966 | 27% | 1036 | 6% | 1256 | 7% | 18683 |
| 5 | 11155 | 62% | 4721 | 26% | 1079 | 6% | 1072 | 6% | 18027 |
| 6 | 12347 | 63% | 5277 | 27% | 1059 | 5% | 1010 | 5% | 19693 |
| 7 | 8476 | 57% | 4565 | 30% | 1030 | 7% | 926 | 6% | 14997 |
| 8 | 7553 | 58% | 3766 | 29% | 969 | 7% | 710 | 5% | 12998 |
| 9 | 7388 | 59% | 3636 | 29% | 895 | 7% | 641 | 5% | 12560 |
| 10 | 6576 | 58% | 3269 | 29% | 920 | 8% | 646 | 6% | 11411 |
| 11 | 6384 | 59% | 3135 | 29% | 790 | 7% | 536 | 5% | 10845 |
| 12 | 9436 | 61% | 4451 | 29% | 952 | 6% | 633 | 4% | 15472 |
| Total | 127891 | 52% | 56803 | 23% | 10870 | 4% | 13930 | 6% | 245324 |
