# Supplementary material for: Patterns of engagement in care during clients’ first 12 months after HIV treatment initiation in South Africa: A retrospective cohort analysis using routinely collected data
Source: PLOS Glob Public Health. 2024 Feb 28;4(2):e0002956. doi: 10.1371/journal.pgph.0002956 (PMC10901315; doi:10.1371/journal.pgph.0002956)
Supplement: S2 Table — (DOCX) [file pgph.0002956.s002.docx]

**S2 Table. Classification of engagement profiles district and year of initiation**

| Outcome | **Enhlanzeni** | | | | | **King Cetswhayo** | | | | | **West Rand** | | | | |
| --- | --- | --- | --- | --- | --- | --- | --- | --- | --- | --- | --- | --- | --- | --- | --- |
|  | 2018 | 2019 | 2020 | 2021 | 2022 | 2018 | 2019 | 2020 | 2021 | 2022 | 2018 | 2019 | 2020 | 2021 | 2022 |
| Total N | (N=3638) | (N=5225) | (N=4275) | (N=3123) | (N=1061) | (N=3099) | (N=3210) | (N=2116) | (N=1679) | (N=649) | (N=2097) | (N=1906) | (N=1615) | (N=1628) | (N=509) |
| **Outcome months 0-6** | |  |  |  |  |  |  |  |  |  |  |  |  |  |  |
| Continuous | 1914 (52.6%) | 3098 (59.3%) | 2452 (57.4%) | 1759 (56.3%) | 581 (54.8%) | 1577 (50.9%) | 2043 (63.6%) | 1464 (69.2%) | 1098 (65.4%) | 405 (62.4%) | 1238  (59.0%) | 1103 (57.9%) | 932 (57.7%) | 1033 (63.5%) | 308 (60.5%) |
| Cyclical | 592 (16.3%) | 528 (10.1%) | 422 (9.9%) | 419 (13.4%) | 162 (15.3%) | 629 (20.3%) | 530 (16.5%) | 270  (12.8%) | 264 (15.7%) | 116 (17.9%) | 259 (12.4%) | 284 (14.9%) | 210 (13.0%) | 201 (12.3%) | 81 (15.9%) |
| Disengaged | 665 (18.3%) | 627 (12.0%) | 773 (18.1%) | 561 (18.0%) | 222 (20.9%) | 498 (16.1%) | 247 (7.7%) | 169 (8.0%) | 143 (8.5%) | 40 (6.2%) | 472 (22.5%) | 396 (20.8%) | 388 (24.0%) | 336 (20.6%) | 96 (18.9%) |
| TFO | 412 (11.3%) | 901 (17.2%) | 573 (13.4%) | 357 (11.4%) | 89 (8.4%) | 365 (11.8%) | 351 (10.9%) | 188 (8.9%) | 152 (9.1%) | 80 (12.3%) | 106 (5.1%) | 105 (5.5%) | 69 (4.3%) | 45 (2.8%) | 20 (3.9%) |
| Death | 55 (1.5%) | 71 (1.4%) | 55 (1.3%) | 27 (0.9%) | 7 (0.7%) | 30 (1.0%) | 39 (1.2%) | 25 (1.2%) | 22 (1.3%) | 8 (1.2%) | 22 (1.0%) | 18 (0.9%) | 16 (1.0%) | 13 (0.8%) | 4 (0.8%) |
| **Outcome months 7-12** | |  |  |  |  |  |  |  |  |  |  |  |  |  |  |
| Continuous | 1512 (41.6%) | 2478 (47.4%) | 1821 (42.6%) | 1359 (43.5%) | 437 (41.2%) | 1272 (41.0%) | 1540 (48.0%) | 1146 (54.2%) | 777 (46.3%) | 308 (47.5%) | 957 (45.6%) | 838 (44.0%) | 738 (45.7%) | 789 (48.5%) | 254 (49.9%) |
| Cyclical | 611 (16.8%) | 511 (9.8%) | 611 (14.3%) | 445 (14.2%) | 175 (16.5%) | 653 (21.1%) | 636 (19.8%) | 339 (16.0%) | 397 (23.6%) | 143 (22.0%) | 292 (13.9%) | 319 (16.7%) | 247 (15.3%) | 276 (17.0%) | 86 (16.9%) |
| Disengaged 1st 6 | 665 (18.3%) | 627 (12.0%) | 773 (18.1%) | 561 (18.0%) | 222 (20.9%) | 498 (16.1%) | 247 (7.7%) | 169 (8.0%) | 143 (8.5%) | 40 (6.2%) | 472 (22.5%) | 396 (20.8%) | 388 (24.0%) | 336 (20.6%) | 96 (18.9%) |
| Disengaged | 217 (6.0%) | 318 (6.1%) | 263 (6.2%) | 271 (8.7%) | 88 (8.3%) | 119 (3.8%) | 163 (5.1%) | 94 (4.4%) | 81 (4.8%) | 34 (5.2%) | 190 (9.1%) | 188 (9.9%) | 135 (8.4%) | 138 (8.5%) | 35 (6.9%) |
| Transferred | 565 (15.5%) | 1195 (22.9%) | 737 (17.2%) | 454 (14.5%) | 128 (12.1%) | 516 (16.7%) | 569 (17.7%) | 334 (15.8%) | 254 (15.1%) | 114 (17.6%) | 159 (7.6%) | 139 (7.3%) | 91 (5.6%) | 72 (4.4%) | 34 (6.7%) |
| Death | 68 (1.9%) | 96 (1.8%) | 70 (1.6%) | 33 (1.1%) | 11 (1.0%) | 41 (1.3%) | 55 (1.7%) | 34 (1.6%) | 27 (1.6%) | 10 (1.5%) | 27 (1.3%) | 26 (1.4%) | 16 (1.0%) | 17 (1.0%) | 4 (0.8%) |
| **Median Number of visits per client** | | |  |  |  |  |  |  |  |  |  |  |  |  |  |
| Median [Q1, Q3] | 7.00 [3.00, 10.0] | 7.00 [3.00, 10.0] | 6.00 [2.00, 8.00] | 6.00 [2.00, 9.00] | 6.00 [3.00, 9.00] | 10.0 [3.00, 12.0] | 10.0 [5.00, 12.0] | 8.00 [5.00, 10.0] | 8.00 [5.00, 10.0] | 8.00 [5.00, 10.0] | 7.00 [3.00, 10.0] | 7.00 [3.00, 9.00] | 7.00 [3.00, 9.00] | 7.00 [4.00, 9.00] | 7.00 [4.00, 10.0] |
