# Supplementary material for: Patterns of engagement in care during clients’ first 12 months after HIV treatment initiation in South Africa: A retrospective cohort analysis using routinely collected data
Source: PLOS Glob Public Health. 2024 Feb 28;4(2):e0002956. doi: 10.1371/journal.pgph.0002956 (PMC10901315; doi:10.1371/journal.pgph.0002956)
Supplement: S3 Table — (DOCX) [file pgph.0002956.s003.docx]

**S3 Table: Classification of engagement patterns during the first and second six months after ART initiation stratified by gender**

| Outcome | Female | Male |
| --- | --- | --- |
|  | (N=23,607) | (N=12,223) |
| Outcome in 1st 6 months | | |
| Continuous | 13713 (58.1%) | 7292 (59.7%) |
| Cyclical | 3316 (14.0%) | 1651 (13.5%) |
| Immediate | 2198 (9.3%) | 1182 (9.7%) |
| Early | 365 (1.5%) | 174 (1.4%) |
| Late | 1166 (4.9%) | 548 (4.5%) |
| Transferred | 2655 (11.2%) | 1158 (9.5%) |
| Died | 194 (0.8%) | 218 (1.8%) |
| Outcome in 2nd 6 months | | |
| Continuous | 10568 (44.8%) | 5658 (46.3%) |
| Cyclical | 3827 (16.2%) | 1914 (15.7%) |
| Disengaged 0-6 | 3729 (15.8%) | 1904 (15.6%) |
| Disengaged 7-12 | 1482 (6.3%) | 852 (7.0%) |
| Transferred | 3753 (15.9%) | 1608 (13.2%) |
| Died | 248 (1.1%) | 287 (2.3%) |
| Number of visits (median, IQR) | 7.00 [3.00, 10.0] | 7.00 [3.00, 10.0] |
| Days between initiation and last visit or disengagement (median, IQR) | 381 [168, 410] | 382 [175, 410] |
