# Supplementary material for: Patterns of engagement in care during clients’ first 12 months after HIV treatment initiation in South Africa: A retrospective cohort analysis using routinely collected data
Source: PLOS Glob Public Health. 2024 Feb 28;4(2):e0002956. doi: 10.1371/journal.pgph.0002956 (PMC10901315; doi:10.1371/journal.pgph.0002956)
Supplement: S4 Table — (DOCX) [file pgph.0002956.s004.docx]

**S4 Table: Characteristics of participants stratified by engagement pattern months 7-12**

|  | Outcome months 7-12 | | | | | | |
| --- | --- | --- | --- | --- | --- | --- | --- |
|  | Continuous | Cyclical | Death | Disengaged | Disengaged 1st 6 | TFO | Overall |
|  | (N=16,226) | (N=5,741) | (N=535) | (N=2,334) | (N=5,633) | (N=5,361) | (N=35,830) |
| **Outcome months 0-6** |  |  |  |  |  |  |  |
| Continuous | 14,238 (87.7%) | 3,866 (67.3%) | 91 (17.0%) | 1,648 (70.6%) | 0 (0%) | 1,162 (21.7%) | 21.005 (58.6%) |
| Cyclical | 1,988 (12.3%) | 1,875 (32.7%) | 32 (6.0%) | 686 (29.4%) | 0 (0%) | 386 (7.2%) | 4,967 (13.9%) |
| Immediate | 0 (0%) | 0 (0%) | 0 (0%) | 0 (0%) | 3,380 (60.0%) | 0 (0%) | 3,380 (9.4%) |
| Early | 0 (0%) | 0 (0%) | 0 (0%) | 0 (0%) | 539 (9.6%) | 0 (0%) | 539 (1.5%) |
| Late | 0 (0%) | 0 (0%) | 0 (0%) | 0 (0%) | 1,714 (30.4%) | 0 (0%) | 1,714 (4.8%) |
| Transferred | 0 (0%) | 0 (0%) | 0 (0%) | 0 (0%) | 0 (0%) | 3,813 (71.1%) | 3,813 (10.6%) |
| Death | 0 (0%) | 0 (0%) | 412 (77.0%) | 0 (0%) | 0 (0%) | 0 (0%) | 412 (1.1%) |
| **Age** |  |  |  |  |  |  |  |
| Median [Min, Max] | 34 [18, 85] | 31 [18, 83] | 39 [18, 85] | 31 [18, 83] | 31 [18, 84] | 30[18, 80] | 32 [18, 85] |
| **Sex** |  |  |  |  |  |  |  |
| Female | 10,568 (65.1%) | 3,827 (66.7%) | 248 (46.4%) | 1,482 (63.5%) | 3,729 (66.2%) | 3,753 (70.0%) | 23607 (65.9%) |
| Male | 5,658 (34.9%) | 1,914 (33.3%) | 287 (53.6%) | 852 (36.5%) | 1,904 (33.8%) | 1,608 (30.0%) | 12223 (34.1%) |
| **CD4 count at ART initiation** |  |  |  |  |  |  |  |
| CD4 <200 at baseline | 3,645 (22.5%) | 1,031 (18.0%) | 223 (41.7%) | 421 (18.0%) | 826 (14.7%) | 929 (17.3%) | 7,075 (19.7%) |
| CD4 200+ at baseline | 8,014 (49.4%) | 2,649 (46.1%) | 97 (18.1%) | 1,143 (49.0%) | 2,315 (41.1%) | 2,583 (48.2%) | 16,801 (46.9%) |
| No baseline CD4 | 4,567 (28.1%) | 2,061 (35.9%) | 215 (40.2%) | 770 (33.0%) | 2,492 (44.2%) | 1,849 (34.5%) | 11,954 (33.4%) |
| **Viral load status at initiation** |  |  |  |  |  |  |  |
| Not done | 15,432 (95.1%) | 5427 (94.5%) | 513 (95.9%) | 2211 (94.7%) | 5377 (95.5%) | 5117 (95.4%) | 34,077 (95.1%) |
| Suppressed | 348 (2.1%) | 136 (2.4%) | 4 (0.7%) | 35 (1.5%) | 88 (1.6%) | 80 (1.5%) | 691 (1.9%) |
| Unsuppressed | 396 (2.4%) | 164 (2.9%) | 17 (3.2%) | 77 (3.3%) | 144 (2.6%) | 146 (2.7%) | 944 (2.6%) |
| Viremic episode | 50 (0.3%) | 14 (0.2%) | 1 (0.2%) | 11 (0.5%) | 24 (0.4%) | 18 (0.3%) | 118 (0.3%) |
| **TB status** |  |  |  |  |  |  |  |
| No | 15118 (93.2%) | 5349 (93.2%) | 423 (79.1%) | 2183 (93.5%) | 5443 (96.6%) | 5124 (95.6%) | 33640 (93.9%) |
| Yes | 1108 (6.8%) | 392 (6.8%) | 112 (20.9%) | 151 (6.5%) | 190 (3.4%) | 237 (4.4%) | 2190 (6.1%) |
