# Supplementary material for: Patterns of engagement in care during clients’ first 12 months after HIV treatment initiation in South Africa: A retrospective cohort analysis using routinely collected data
Source: PLOS Glob Public Health. 2024 Feb 28;4(2):e0002956. doi: 10.1371/journal.pgph.0002956 (PMC10901315; doi:10.1371/journal.pgph.0002956)
Supplement: S5 Table — (DOCX) [file pgph.0002956.s005.docx]

**S5 Table: Crude and adjusted predictors of becoming disengaged from care**

| **Characteristic** | **Measure** | **Disengaged months 0-6 (n=5,633)** | | **Disengaged months 7-12* (n=2,334)** | | |
| --- | --- | --- | --- | --- | --- | --- |
|  |  | **Crude RR (95% CI)** | **Adjusted RR (95% CI)** | | **Crude RR (95% CI)** | **Adjusted RR**† **(95% CI)** |
| 6-month outcome | Continuous | -- | -- | | ref. | ref. |
|  | Cyclical | -- | -- | | 1.76 (1.62-1.91) | 1.75 (1.61-1.91) |
| Age (years) | 18-25 | 1.73 (1.54-1.94) | 1.85 (1.65-2.08) | | 2.06 (1.70-2.51) | 2.13 (1.75-2.59) |
|  | 26-49 | 1.37 (1.23-1.53) | 1.43 (1.28-1.59) | | 1.63 (1.35-1.96) | 1.65 (1.38-1.98) |
|  | >50 | ref. | ref. | | ref. | ref. |
| Sex | Female | ref. | ref. | | ref. | ref. |
|  | Male | 0.98 (0.94-1.04) | 1.09 (1.03-1.15) | | 1.09 (1.01-1.19) | 1.22 (1.12-1.33) |
| District | Mpumalanga | ref. | ref. | | ref. | ref. |
|  | KwaZulu-Natal | 0.62 (0.58-0.66) | 0.62 (0.58-0.66) | | 0.60 (0.54-0.67) | 0.58 (0.53-0.64) |
|  | Gauteng | 1.32 (1.25-1.40) | 1.27 (1.20-1.34) | | 1.25 (1.14-1.37) | 1.27 (1.16-1.39) |
| Year of ART initiation | 2018 | ref. | ref. | | ref. | ref. |
|  | 2019 | 0.66 (0.62-0.71) | 0.74 (0.69-0.79) | | 1.04 (0.93-1.16) | 1.08 (0.97-1.2) |
|  | 2020 | 0.89 (0.84-0.96) | 0.97 (0.90-1.03) | | 1.01 (0.90-1.14) | 1.03 (0.91-1.16) |
|  | 2021 | 0.87 (0.81-0.94) | 0.92 (0.86-0.99) | | 1.21 (1.08-1.36) | 1.19 (1.06-1.34) |
|  | 2022 | 0.87 (0.79-0.97) | 0.92 (0.83-1.02) | | 1.12 (0.95-1.33) | 1.10 (0.93-1.31) |
| Baseline CD4 | <200 | ref. | ref. | | ref. | ref. |
|  | 200+ | 1.18 (1.09-1.27) | 1.16 (1.08-1.25) | | 1.15 (1.04-1.29) | 1.18 (1.06-1.32) |
|  | No baseline CD4 | 1.79 (1.66-1.92) | 1.62 (1.50-1.74) | | 1.25 (1.12-1.40) | 1.10 (0.98-1.24) |
| Prior ART use | Naïve | ref | ref. | | ref. | ref. |
|  | PMTCT | 1.01 (0.91-1.10) | 0.86 (0.78-0.95) | | 1.34 (1.17-1.53) | 1.23 (1.07-1.41) |
|  | PMTCT and Prior ART | 0.88 (0.49-1.56) | 0.77 (0.43-1.37) | | 0.93 (0.36-2.38) | 0.82 (0.32-2.11) |
|  | Prior ART >30 days (EXP) | 1.42 (1.18-1.69) | 1.20 (1.00-1.42) | | 1.25 (0.91-1.73) | 1.09 (0.79-1.51) |
| On TB treatment at ART initiation | No | ref | ref. | | ref. | ref. |
|  | Yes | 0.45 (0.39-0.52) | 0.47 (0.41-0.55) | | 0.81 (0.69-0.96) | 0.97 (0.81-1.15) |
| WHO disease classification at ART initiation | Stage 1 | ref. | ref. | | ref. | ref. |
|  | Stage 2 | 1.02 (0.93-1.11) | 1.05 (0.97-1.14) | | 0.92 (0.81-1.06) | 0.99 (0.86-1.14) |
|  | Stage 3 | 0.83 (0.72-0.94) | 1.04 (0.91-1.20) | | 1.01 (0.84-1.23) | 1.10 (0.90-1.36) |
|  | Stage 4 | 0.86 (0.62-1.20) | 0.89 (0.65-1.23) | | 1.03 (0.65-1.65) | 1.05 (0.66-1.69) |
|  | Unknown | 1.19 (1.07-1.33) | 1.17 (1.05-1.30) | | 0.79 (0.64-1.00) | 0.87 (0.70-1.08) |
