# Supplementary material for: Patterns of engagement in care during clients’ first 12 months after HIV treatment initiation in South Africa: A retrospective cohort analysis using routinely collected data
Source: PLOS Glob Public Health. 2024 Feb 28;4(2):e0002956. doi: 10.1371/journal.pgph.0002956 (PMC10901315; doi:10.1371/journal.pgph.0002956)
Supplement: S6 Table — (DOCX) [file pgph.0002956.s006.docx]

**S6 Table:** **Sensitivity analysis adjusting for potential outcome misclassification of the effect of engagement pattern at 6 months on ART on risk of disengagement from care during months 7-12**

| **Analysis** | **Crude relative risk* (95% CI)** |
| --- | --- |
| **Conventional analysis (analyze data as observed)** | **1.84 (1.70-1.99)** |
| **Data adjusted for outcome misclassification:**  1. Assume non-differential outcome misclassification of 8% for both continuous and cyclical engagers | 1.95 (1.80 – 2.11) |
| 2. Assume non-differential outcome misclassification of 26% for both continuous and cyclical engagers | 1.95 (1.81 – 2.09) |

***** Continuous engagers are reference group for all RR presented
