# Supplementary material for: Patterns of engagement in care during clients’ first 12 months after HIV treatment initiation in South Africa: A retrospective cohort analysis using routinely collected data
Source: PLOS Glob Public Health. 2024 Feb 28;4(2):e0002956. doi: 10.1371/journal.pgph.0002956 (PMC10901315; doi:10.1371/journal.pgph.0002956)
Supplement: S7 Table — (DOCX) [file pgph.0002956.s007.docx]

**S7 Table: ART regimen by year of initiation**

|  | 2018 | 2019 | 2020 | 2021 | 2022 | Overall |
| --- | --- | --- | --- | --- | --- | --- |
|  | (N=8,834) | (N=10,341) | (N=8,006) | (N=6,430) | (N=2,219) | (N=35,830) |
| Drug regimen line initiated on |  |  |  |  |  |  |
| First | 8,816 (99.8%) | 10,328 (99.9%) | 7,996 (99.9%) | 6,428 (100.0%) | 2,218 (100.0%) | 35,786 (99.9%) |
| Second | 18 (0.2%) | 11 (0.1%) | 10 (0.1%) | 2 (0.0%) | 1 (0.0%) | 42 (0.1%) |
| Third | 0 (0%) | 2 (0.0%) | 0 (0%) | 0 (0%) | 0 (0%) | 2 (0.0%) |
| Initiated on a Dolutegravir-based regimen |  |  |  |  |  |  |
| No | 8,780 (99.4%) | 10,142 (98.1%) | 3,600 (45.0%) | 1,270 (19.8%) | 81 (3.7%) | 23,873 (66.6%) |
| Yes | 54 (0.6%) | 199 (1.9%) | 4,406 (55.0%) | 5,160 (80.2%) | 2,138 (96.3%) | 11,957 (33.4%) |
| ART regimen initiated on |  |  |  |  |  |  |
| TDF/3TC/DTG | 52 (0.6%) | 188 (1.8%) | 4,326 (54.0%) | 5,101 (79.3%) | 2,104 (94.8%) | 11,771 (32.9%) |
| TDF/FTC/EFV | 8,645 (97.9%) | 10,034 (97.0%) | 3,538 (44.2%) | 1,239 (19.3%) | 74 (3.3%) | 23,530 (65.7%) |
| Other | 137 (1.6%) | 119 (1.2%) | 142 (1.8%) | 90 (1.4%) | 41 (1.8%) | 529 (1.5%) |
| ART regimen at last observed visit |  |  |  |  |  |  |
| TDF/3TC/DTG | 57 (0.6%) | 1,255 (12.1%) | 4,838 (60.4%) | 5,353 (83.3%) | 2,129 (95.9%) | 13,632 (38.0%) |
| TDF/FTC/EFV | 8,537 (96.6%) | 8,778 (84.9%) | 2,978 (37.2%) | 854 (13.3%) | 37 (1.7%) | 21,184 (59.1%) |
| Other | 240 (2.7%) | 308 (3.0%) | 190 (2.4%) | 223 (3.5%) | 53 (2.4%) | 1,014 (2.8%) |
